# Establishment of a sensitive UPLC-MS/MS method to quantify safinamide in rat plasma

**DOI:** 10.3389/fphar.2023.1211383

**Published:** 2023-08-28

**Authors:** Ying Wang, Guan-An Zhao, Xia Li, En Zhang, Wei Tan, Jia-qi Chen

**Affiliations:** ^1^ Department of Pharmacy, The Affiliated Lihuili Hospital, Ningbo University, Ningbo, Zhejiang, China; ^2^ Urinary Surgery, The People’s Hospital of Lishui, Lishui, Zhejiang, China; ^3^ Clinical Laboratory, The Affiliated Lihuili Hospital, Ningbo University, Ningbo, Zhejiang, China; ^4^ The Third Affiliated Hospital of Chongqing Medical University (Gener Hospital), Chongqing, China; ^5^ Clinical Lab, The People’s Hospital of Lishui, Lishui, Zhejiang, China

**Keywords:** UPLC-MS/MS, rat, safinamide, plasma, pharmacokinetics

## Abstract

A fast, simple, and sensitive ultra performance liquid chromatography tandem mass spectrometry (UPLC-MS/MS) method was established for the quantification of safinamide in rat plasma. Plasma samples were treated with acetonitrile for protein precipitation, and diazepam was used as an internal standard (IS). The analytes were separated on an Acquity UPLC C18 (2.1 mm × 50 mm, 1.7 μm) chromatographic column with gradient elution using a mobile phase (0.1% formic acid-acetonitrile). Then, the eluates were detected by electrospray ionization (ESI) in positive ion mode. The analytes were quantified by multiple reaction monitoring (MRM) using the transition m/z 303.3→215.0 of safinamide and m/z 285.0→154.0 of IS. Safinamide had good linearity in the concentration range of 1.0–2000 ng/mL, and the lower limit of quantitation (LLOQ) was 1.0 ng/mL. The intra- and inter-day precision and accuracy of safinamide were less than 7.63%, while the average recovery rate was 92.98%–100.29%. The method was validated to be stable and had low noise, short chromatographic run time, wide linear range, small sample volumes, low sample injection volumes, and high sensitivity. Therefore, it can be used in pharmacokinetics and preclinical and clinical studies.

## 1 Introduction

Safinamide ((2S)-2-[[4-[(3-fluorophenyl) methoxy] phenyl] methylamino] propanamide), used to clinically treat Parkinson’s disease (PD), is a novel, reversible, and highly selective monoamine oxidase B (MAO-B) inhibitor (Fariello et al., 1998; Maj et al., 1998; Maj et al., 1999; Muller, 2017; Hattori et al., 2020; Müller, 2020; Rinaldi et al., 2021; Stocchi et al., 2021). The relative contributing mechanism of safinamide may be relevant to two properties: dopaminergic properties (highly selective, reversible MAO-B) and non-dopaminergic properties (selective sodium channel blockade, selective calcium channel modulation, and consequent excessive glutamate release inhibition) (Salvati et al., 1999; Fariello et al., 2000; Fariello, 2007; Deeks, 2015; Desouza and Schapira, 2017).

Safinamide is a water-soluble drug for once-daily administration. It is absorbed very quickly, and the peak plasma concentration is in the range of 2–4 h. It displays complete and reliable absorption and showed a terminal half-life period of approximately 26 h, with a total bioavailability of 95% (Krosser et al., 2012; Leuratti et al., 2013). Food might prolong the rate of safinamide but does not affect the extent of absorption. After repeated dosing once daily, safinamide homeostasis was achieved on day 5 of treatment, with a cumulative factor of 1.5–1.7.

As an alternative to levodopa, safinamide had been approved for the treatment of PD in Europe and the United States (Di Stefano and Rusca, 2011; Teixeira et al., 2018). It is considered to be a new hope for PD, but still needs trials or studies to prove its potential as an anti-PD drug. However, few methodologies have been established to study this medicine in biological fluids. One study reported that safinamide in humans and various animals was detected by HPLC-MS/MS or HPLC-FD using three methods (Dal Bo et al., 2006). These methods are not widely available to others due to elaborate sample preparation (liquid-liquid extraction), large sample volume (up to 0.5 mL), and low sensitivity (20 ng/mL). Recently, a chiral HPLC method with low sensitivity and long analytical run time (15 min) was developed to detect the (R)-enantiomer in safinamide mesilate (Zhang et al., 2011). To solve the above deficiencies, we have developed a simple and efficient ultra performance liquid chromatography tandem mass spectrometry (UPLC-MS/MS) method with an LLOQ of 1.0 ng/mL for safinamide.

## 2 Materials and methods

### 2.1 Chemicals and reagents

**FIGURE 1:**
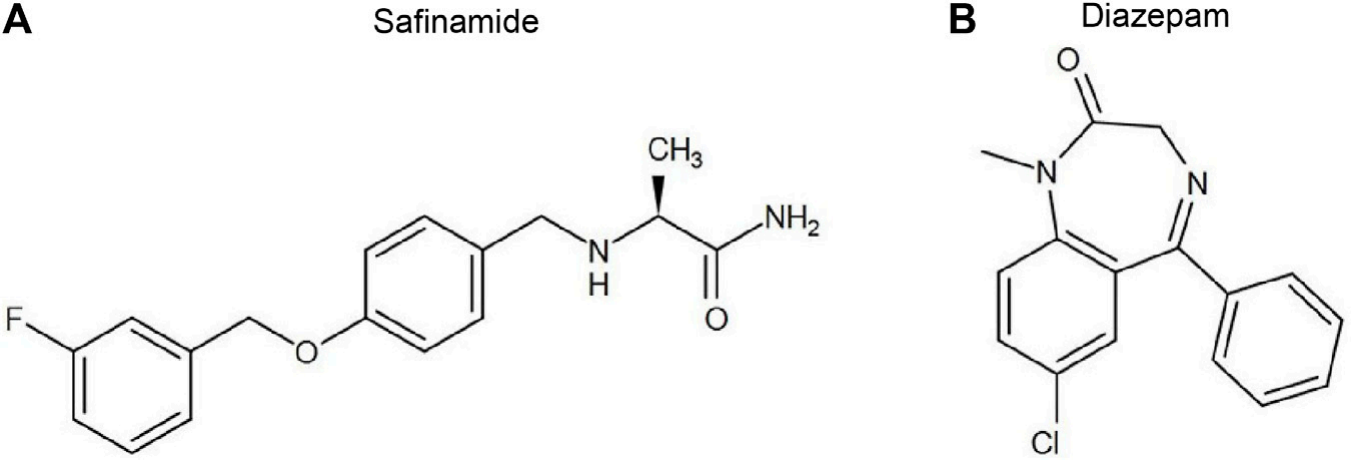
Chemical structures of safinamide **(A)** and diazepam **(B)**.

Safinamide mesylate (CAS NO: 133,865-89-1) (Figure 1A) and diazepam (CAS NO: 65854-76-4) as an internal standard (IS) (Figure 1B) were obtained from Beijing sunflower and technology Development CO., LTD and Sigma-Aldrich Company (St. Louis, MO, United States), respectively. Formic acid (CAS NO: 64-18-6), LC grade acetonitrile, and methanol were purchased from Anaqua Chemicals Supply (ACS, American) and Merck KGaA (Darmstadt, Germany), respectively. Other reagents were of analytical grade. The experimental water was prepared using a Millipore (Bedford, MA) Milli-Q A10 purification system. The materials used in the experiment met the requirements of LC/MS or UPLC/MS.

### 2.2 UPLC-MS/MS conditions

A Waters Acquity UPLC I-Class and a Waters XEVO TQ-S tandem quadrupole mass spectrometer (Waters, Milford, United States) constituted the UPLC-MS/MS equipment. An Acquity UPLC BEH C18 (2.1 mm × 50 mm, 1.7 μm; Waters, Milford, United States) column (temperature 40°C) was used to separate the analytes after 2 μL of extracted sample was injected. The mobile phase consisted of solution A (0.1% formic acid) and solution B (acetonitrile), with the following conditions: 0–0.5 min 10% B; 0.5–1.0 min 10%–90% B; 1.0–2.0 min 90% B; 2.0–2.1 min 90%–10% B; 2.1–3.0 min 10% B. Safinamide and IS were eluted at 1.21 min and 1.41 min, respectively. Under a flow rate of 0.4 mL/min, the chromatographic run time was 3.0 min.

Identification was performed in the positive electrospray ionization (ESI^+^) mode. The ion source was set as follows: capillary voltage 3.0 kV, source temperature 150 °C, desolvation temperature 800 °C, cone gas 150 L/h, and desolvation gas 650 L/h. Multiple reaction monitoring (MRM) mode was used for quantification according to the target fragment ions of *m/z* 303.3→215.0 for safinamide and *m/z* 285.0→154.0 for IS. MassLynx V4.1 software (Waters, Milford, United States) was used for system operation, data acquisition, and data processing.

### 2.3 Calibration standards

Individual stock solutions of safinamide and IS in LC grade methanol were prepared at a concentration of 1.0 mg/mL. The stock solutions were diluted into calibration and control solutions, respectively. The IS working standard solution was prepared by diluting the reserve solution in methanol at a concentration of 500 ng/mL. Blank rat plasma was spiked with the appropriate working solution amounts to prepare the safinamide calibration standard. The calibration plot of safinamide in rat plasma was linear in the range 1.0–2000 ng/mL (1.0, 5.0, 10, 50, 100, 500, 1,000, and 2000 ng/mL). As calibration standards (2.0, 800, and 1,600 ng/mL), the quality control (QC) samples were prepared independently in the same way. All solutions were stored in a refrigerator at 4°C and were brought to room temperature before use.

### 2.4 Sample preparation

The frozen plasma sample was thawed at room temperature before analysis. The 100 μL plasma sample containing 20 μL IS (500 ng/mL) was added to 300 μL acetonitrile in a 1.5 mL centrifuge tube. The tube was vortexed for 2.0 min before centrifugation at 13,000 rpm for 15 min. Then, the supernatant (2 μL) was injected into the UPLC-MS/MS system for further analysis.

### 2.5 Method validation

The selectivity, precision, accuracy, recovery, and stability were approved for the analytical method according to FDA guidelines (Xu et al., 2019; Tang et al., 2020). Selectivity was usually assessed by the degree of interference from the peaks of safinamide and IS at their retention times.

Three different concentrations (2.0, 800, and 1,600 ng/mL) of QC samples were used to assess the precision and accuracy. Examination of QC samples (n = 6) at different times in 1 day was performed to evaluate the intra-day accuracy and precision. QC sample assays were repeated for three consecutive days to evaluate the inter-day accuracy and precision. Relative error (RE) was used to estimate the accuracy, while relative standard deviation (RSD) was used to evaluate the precision. The recovery rate was calculated by comparing the peak area of extracted plasma samples with the peak area obtained by directly injecting the corresponding concentration of standard solution in the extracted blank plasma at three different concentrations (2.0, 800, and 1,600 ng/mL). After protein precipitation, 2.0, 800, and 1,600 ng/mL of analytes were added to blank rat plasma to evaluate matrix effects. Exposed to different conditions, five replicates of plasma samples (2.0, 800, and 1,600 ng/mL) were analyzed to evaluate the stability of safinamide in rat plasma. After exposure to two different conditions (room temperature and 4°C) for 12 h, the spiked samples were examined to evaluate the short-term stability. After three consecutive complete freeze/thaw cycles (−20°C–25°C), the spiked samples were studied to assess the freeze/thaw stability. The spiked samples stored at −80°C for 28 days were measured to evaluate the long-term stability.

### 2.6 Pharmacokinetic study

Eight male Sprague-Dawley rats (180–220 g) purchased from the Laboratory Animal Center of The First Affiliated Hospital of Wenzhou Medical University (Zhejiang, China) were used to study the pharmacokinetics of safinamide. All experimental procedures and protocols were operated under the National Institutes of Health Guide for the Care and Use of Laboratory Animals and were approved by the Animal Care and Use Committee of The First Affiliated Hospital of Wenzhou Medical University before the study began.

Water was freely available throughout the experiment but the rats were fasted for 12 h before the experiment. Safinamide dissolved in 0.5% sodium carboxymethyl cellulose (2 mg/mL) was orally given to eight young adult Sprague-Dawley rats (180–220 g, 10 mg/kg). Heparinized 1.5 mL polythene tubes were used to collect 0.3 mL blood samples from the tail vein at different time points (0.33, 0.67, 1, 1.5, 2, 3, 4, 6, 9, 12, and 24 h) after oral safinamide. After centrifuging at 4,000 g for 8 min, 100 µL of the supernatant plasma was separated and stored at −80°C until analysis. The safinamide data of plasma concentration *versus* time were processed and analyzed using DAS (drug and statistics) software (Version 2.0, Shanghai University of Traditional Chinese Medicine, China).

## 3 Results

### 3.1 Method development and optimization

Sample preparation, chromatographic separation, and appropriate ionization play critical roles in the development of the method. For sample preparation before UPLC-MS/MS analysis, it was important to employ an effective biological sample removal process to remove proteins and potential interferences. The method in this report used a fast, simple, and effective protein precipitation method as the extraction technique, replacing the existing complex liquid-liquid extraction technology reported in other studies. Using acetonitrile as the protein precipitation solvent was more effective than methanol, trichloroacetic acid (10%), or perchloric acid (6%). Meanwhile, acetonitrile also had an acceptable recovery.

Different combinations (acetonitrile, methanol, water, and formic acid in water) and their proportions were examined in this study to determine the optimal mobile phase. When acetonitrile was used as the mobile phase, the peak shape of the analyte was sharper and symmetrical. Therefore, acetonitrile was selected as the organic solvent. To improve the sensitivity of this method, 0.1% formic acid was added to the mobile phase. Gradient elution was adopted because of its better peak symmetry, proper retention time, and minimal matrix effects. Different flow rates were tested and 0.4 mL/min was selected based on the peak shape of the standard and sample. The total run time of the safinamide detection method established in this study was 3.0 min.

The best MRM quantitative mode was *m/z* 303.3→215.0 for safinamide and *m/z* 285.0→154.0 for IS. In addition, bupivacaine, midazolam, metoprolol, and diazepam were tested to obtain a suitable IS. Finally, diazepam stood out from the crowd for its stable ionization and suitable retention time in MRM mode. In addition to these factors, other important variables (including source temperature, carrier gas flow rate, collision gas flow rate, column temperature, injection volume, *etc.*) were also studied to obtain satisfactory corresponding qualitative data.

### 3.2 Selectivity and matrix effect

**FIGURE 2:**
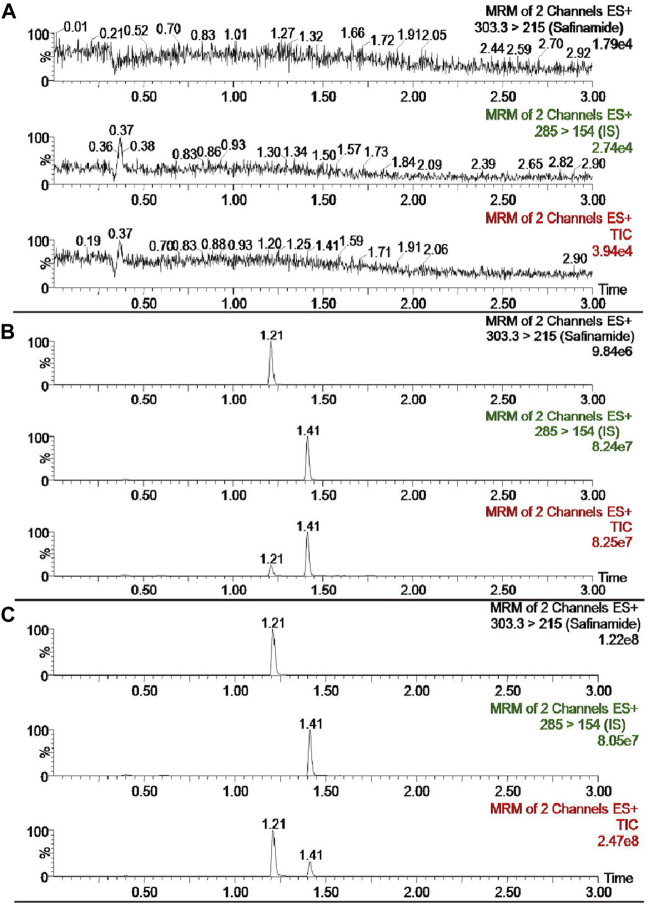
UPLC-MS/MS chromatograms of safinamide and diazepam (IS). **(A)** blank plasma sample; **(B)** blank plasma sample spiked with safinamide and IS. **(C)** real plasma sample from a rat after oral administration of safinamide.

The selectivity of the detection method was evaluated using the chromatograms of the blank plasma, the blank plasma spiked with safinamide and IS, and the real plasma sample after oral administration of safinamide. Figure 2 shows the relevant chromatograms.

The retention period time of the analyte and IS did not show interfering endogenous substances. At concentrations of 2.0, 800, and 1,600 ng/mL, the matrix effects for safinamide were 99.95 ± 14.02, 108.19 ± 10.19, 98.93% ± 3.21% (*n* = 6), respectively. No matrix effects exists when the matrix effect = 100, possible matrix enhancement exists when the matrix effect >100, and possible matrix suppression exists when the matrix effect <100. Therefore, the matrix effect of this method had a negligible influence on the current research.

### 3.3 Calibration curve and sensitivity

The calibration curve of safinamide in rat plasma ranged from 1.0 to 2000 ng/mL. Table 1 summarizes the linearity parameters obtained for safinamide by analyzing the rat plasma calibration curves. The LLOQ of the current UPLC-MS/MS method was 1.0 ng/mL for safinamide. This LLOQ is relatively more sensitive than those obtained in previous methods (20 ng/mL) (Dal Bo et al., 2006; Zhang et al., 2011).

**TABLE 1:** Calibration curve parameters for safinamide in rat plasma (*n* = 3).

Sample Matrix	Calibration range (ng/mL)	Slope	Intercept	*r* ^2^
		Mean ± SD	Mean ± SD	Mean ± SD
Rat plasma	1.0 - 2000	0.0536 ± 0.0022	0.4884 ± 0.0954	0.9958 ± 0.001

### 3.4 Precision, accuracy, and recovery

Blank plasma spiked with safinamide at 2.0, 800, and 1,600 ng/mL was used to assess the precision and accuracy of the entire analytical procedure on the same day or for three consecutive days. At QC levels of 2.0, 800, and 1,600 ng/mL, the intra-day precision was not more than 5.86%, and the inter-day precision was not more than 6.42%. The accuracy of this method ranged from −7.63% to 4.02%. The recoveries of safinamide from rat plasma were better than 92.98%. These results are shown in Table 2. The method was accurate and precise because the values above are within the acceptable ranges.

**TABLE 2:** The precision, accuracy and extraction efficiency of safinamide in rat plasma (*n* = 6).

Compound	Concentration (ng/mL)	Intra-day (ng/mL)	Inter-day (ng/mL)	Extraction efficiency (%)
		Mean ± SD	Mean ± SD	Mean ± SD
Safinamide	2.0	1.94 ± 0.07	1.86 ± 0.12	92.98 ± 7.94
	800	832.19 ± 48.79	801.08 ± 46.93	100.29 ± 6.21
	1,600	1,477.96 ± 38.17	1,521.92 ± 52.30	98.24 ± 2.27

### 3.5 Stability

Under various storage conditions (room temperature, 4°C, three complete freeze-thaw cycles (−20°C–25°C), and −80°C), the stability results of safinamide were stable in rat plasma. The data showed the following: |RE| ≤ 7.43 after 12 h at room temperature, |RE| ≤ 9.18 after 12 h at 4°C, |RE| ≤ 5.66 after three freeze–thaw-cycles in rat plasma, and |RE| ≤ 12.74 after 28 days storage at −80°C. The established method could be used for further pharmacokinetics studies due to its precision (RSD <15%). These results are summarized in Table 3.

**TABLE 3:** Stability of safinamide in rat plasma under various storage conditions (*n* = 5).

Storage conditions	Concentration (ng/mL)	Mean (ng/mL)	RSD (%)	RE (%)
Room temperature/12 h	2.0	2.10	5.26	5.17
	800	859.42	6.23	7.43
	1,600	1,626.98	2.57	1.69
4°C/12 h	2.0	2.01	4.49	0.59
	800	873.46	5.56	9.18
	1,600	1713.90	1.97	7.12
Three complete freeze-thaw cycles (−20°C–25°C)	2.0	2.11	14.81	5.66
	800	841.53	9.93	5.19
	1,600	1,672.28	11.25	4.52
−80°C/28 days	2.0	2.03	7.26	1.56
	800	901.91	8.32	12.74
	1,600	1772.13	9.98	10.76

### 3.6 Application of the method in a pharmacokinetics study

**FIGURE 3:**
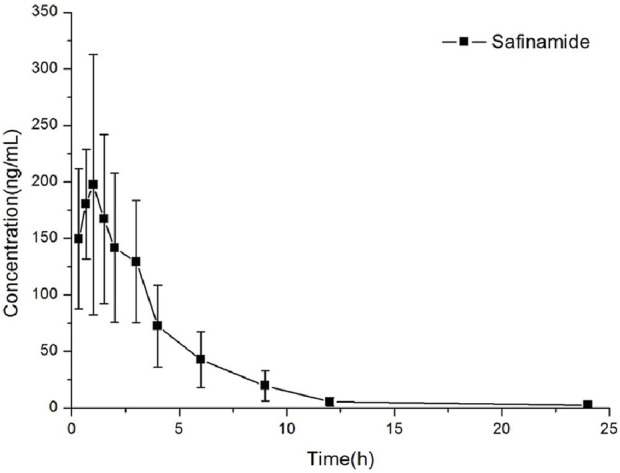
Concentration *versus* time curves of safinamide in rats after a single oral administration at 10 mg/kg.

This method could be effectively used to determine the content of safinamide in the plasma of rats after oral administration (10 mg/kg). The mean plasma concentration–time curve and the main pharmacokinetic parameters (non-compartment model analysis) are shown in Figure 3; Table 4, respectively.

**TABLE 4:** The pharmacokinetic parameters of safinamide in rat plasma after oral administration 10 mg/kg.

Parameters	Mean ± SD
t_1/2_ (h)	1.82 ± 0.38
C_max_ (ng/mL)	256.03 ± 68.99
T_max_ (h)	1.19 ± 0.60
CLz/F (L/h/kg)	13.37 ± 3.89
AUC_0→t_ (ng/mL•h)	793.84 ± 227.51
AUC_0→∞_ (ng/mL•h)	806.795 ± 234.31

## 4 Conclusion

A fast, simple, and novel UPLC-MS/MS method with good linearity in the concentration range of 1.0–2000 ng/mL was developed and successfully used to detect safinamide in rat plasma. Compared to other methods, the current method could meet the requirements of bioavailability studies on safinamide because of its high sensitivity, small sample volume, low injection volume, and short analysis time.

## Data Availability

The original contributions presented in the study are included in the article/Supplementary material, further inquiries can be directed to the corresponding author.
